# Synthesis and Structural Modification of Marine Natural Products

**DOI:** 10.3390/molecules22060882

**Published:** 2017-05-26

**Authors:** Juan Zhang, Hua Zhang, Luis Alexandre Muehlmann, Cheng-Shi Jiang, Yue-Wei Guo

**Affiliations:** 1 School of Biological Science and Technology, University of Jinan, Jinan 250022, China; zjandzq@163.com (J.Z.); bio_zhangh@ujn.edu.cn (H.Z.); 2 Faculdade de Ceilândia, University of Brasília, Brasilia 70910900, Brazil; luisalex@unb.br; 3 Shanghai Institute of Materia Medica, Chinese Academy of Sciences, Shanghai 201203, China

**Keywords:** marine natural products, total synthesis, structural modification, antitumor activity, enzyme-inhibitory activity, neuroprotective activity

## Abstract

In the last decades, marine natural products (MNPs), have attracted extensive interest from both chemists and pharmacologists due to their chemical and bioactive diversities. This special issue, collecting total synthesis and structural modification of six different type of bioactive MNPs, is expected to inspire and attract more research effects invested into MNP research.

## 1. Introduction

It is not surprising that marine organisms can produce secondary metabolites that are very different from those produced by terrestrial plants, given their special marine living environment conditions including high concentration of salts, high pressure, low concentration of oxygen, and dark condition [1]. In recent years, the capability of marine organisms to produce highly potent bioactive metabolites with unique structures has attracted wide interest of chemists and pharmacologists aiming to find marine natural products (MNPs) with potential clinical value [2,3].

In the projects aiming at discovering drug leads/candidates derived from MNPs, a number of novel bioactive MNPs and their analogs have been identified. In this special issue, the total synthesis and structural modification of six different types of bioactive MNPs associated with Guo’s, and other, groups will be discussed.

## 2. The Synthesis and Modification of Bioactive NMPs

### 2.1. Phidianidines A *(**1a**)* and B *(**1b**)*

**Figure 1:**
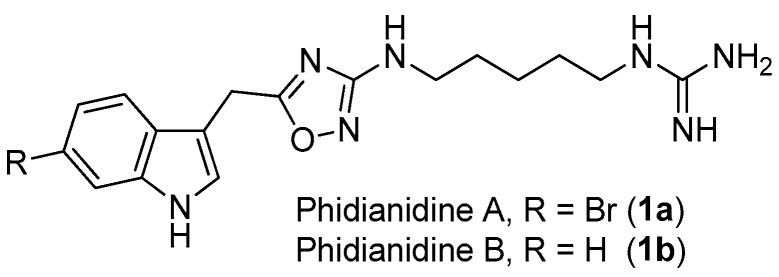
The structures of phidianidines A (**1a**) and B (**1b**).

1,2,4-Oxadiazole is a common, but very important, pharmacophore widely found in synthetic molecules. However, natural products possessing a 1,2,4-oxadiazole ring system are extremely rare. To the best of our knowledge, phidianidines A (**1a**) and B (**1b**) (Figure 1) are the first natural representatives of 1,2,4-oxadiazole derivatives, isolated from Chinese aeolid opisthobranch *Phidiana militaris* [4]. Both novel tryptophan-derived alkaloids showed robust cytotoxicity against C6 and HeLa cells, within nanomolar range. In addition, phidianidine A was found to be a new antagonist of CXCR4 [5], an important therapeutic target for the treatment of diseases like HIV infection, rheumatoid arthritis and cancer.

**Figure 2:**
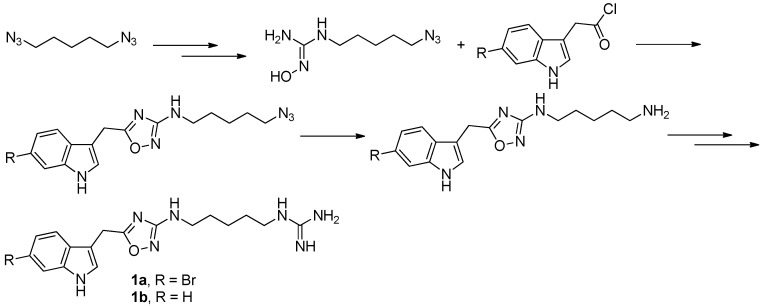
Sinder’s protocol [6] for the synthesis of phidianidines.

**Figure 3:**
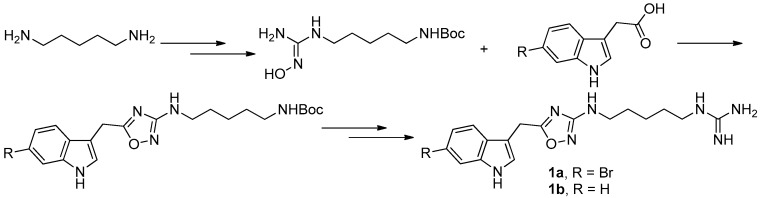
Lindsley’s protocol [7] for the synthesis of phidianidines.

**Figure 4:**
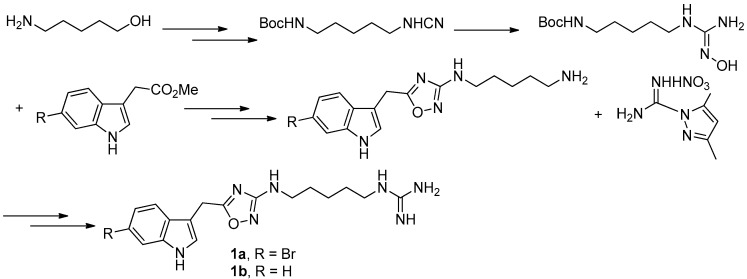
Manzo’s protocol [8] for the synthesis of phidianidines.

**Figure 5:**
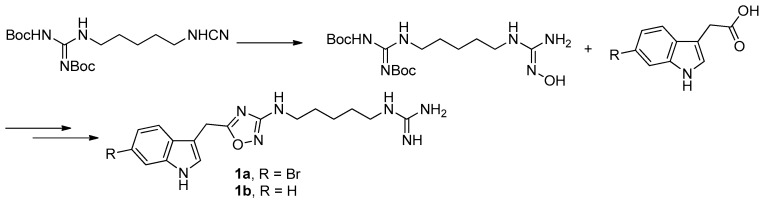
Chamberland’s protocol [9] for the synthesis of phidianidines.

To provide novel phidianidines derivatives for further pharmacological study, several research groups have launched projects aiming at their total syntheses. The first synthesis of phidianidines A (**1a**) and B (**1b**) was reported by Snider et al. (Figure 2) [6]. Generally, the synthesis started with 1,5-diazidopentane and the key 1,2,4-oxadiazole intermediates were generated by the reaction of indole-3-acetyl chloride with *N*-5-azidopentyl-*N*′-hydroxyguanidine. Then, reduction of the terminal azide afforded the corresponding amine, which was smoothly transformed to guanidine. Finally, the syntheses of phidianidines were completed in 19% overall yield. At almost the same time, Lindsley and coworkers described the synthesis of **1a** and **1b** (Figure 3), with a similar approach to that of Snider [7]. In their work, both alkaloids were found to be selective inhibitors of the dopamine transporter (**1a**: IC_50_ = 390 nM; **1b**: IC_50_ = 680 nM) and selective agonists of the μ opioid receptor (**1a**: EC_50_ = 17% nM; **1b**: EC_50_ = 12%). However, there are some critical aspects, such as the use of toxic reagents (i.e., BrCN) and the formation of unstable intermediates in the above protocols. In order to avoid these inconveniences, Manzo and coworkers reported a simpler and easier synthetic route, which is based on the coupling of 3-indolacetic acid methyl ester and the amino-alkyl hydroxy guanidine intermediate (Figure 4) [8]. The latest concise synthetic approach reported by Chamberland et al. was inspired by the proposed biosynthetic hypothesis of phidianidines. In their protocol, the azide was not used as an amine protecting group based on safety concern and the guanidine was installed first to eliminate the unnecessary steps of deprotection (Figure 5) [9].

**Figure 6:**
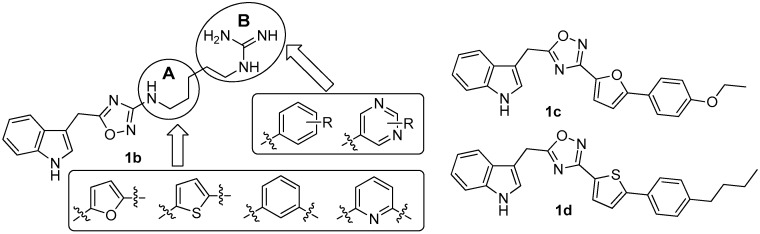
The synthesis of phidianidine B mimic in Guo’s group [10,11].

Since there was no structure-activity relationship (SAR) study on phidianidines, the first mimic synthesis and SAR study of phidianidine B (**1b**) was carried out by Guo’s group (Figure 6). Based on a scaffold hopping strategy, the benzene and other aromatic ring systems were introduced to modify the alkyl-guanidinium side chain, resulting in a series of phidianidine B-based analogs as shown in Figure 6. First, the alkylguanidinium side chain was modified by the introduction of the furan ring moiety in part A and substituted phenyl or aminopyrimidine in part B; the furan ring moiety was then replaced by thiophene, benzene, or pyridine. All of these analogs were evaluated for bioactivities in different screen models. Among these analogs, derivative **1c** showed good in vitro neuroprotective effects against amyloid-β_25–35_-, hydrogenperoxide-, and oxygen-glucose deprivation-induced neurotoxicity in SH-SY5Y cells, indicating that **1c** is a promising drug candidate for treating Alzheimer’s disease [10]. In addition, derivative **1d** was found to be a novel potent selective inhibitor of protein tyrosine phosphatase 1B (PTP1B, IC_50_ = 8.96 µM), a potential target for treating type II diabetes and obesity [11].

### 2.2. Gymnorrhizol *(**2a**)* and Bruguiesulfurol *(**2b**)*

**Figure 7:**
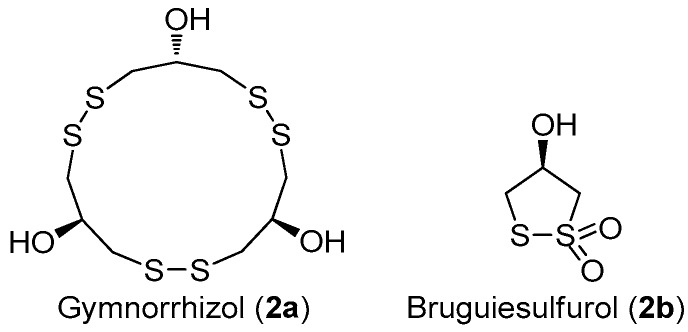
The structures of gymnorrhizol (**2a**) and bruguiesulfurol (**2b**).

Marine disulfide- and multisulfide-containing metabolites are a special and important class of natural products, which exhibited promising bioactivities, including antitumor, antibiotic, and enzyme-inhibitory activities. In particular, the disulfide bond was found to play an important role for their bioactivities [12]. The chemical constituent investigation on the Chinese marine mangrove *Bruguiera gymnorrhiza* resulted in the isolation of several cyclic disulfides [13,14,15,16]. Among these disulfides, an unusual 15-membered macyrocylic polydisulfide, gymnorrhizol (**2a**), and its known monomer, bruguiesulfurol (**2b**) (Figure 7) [17], were first found to be promising PTP1B inhibitors, with IC_50_ values of 14.9 and 17.5 µM, respectively.

**Figure 8:**
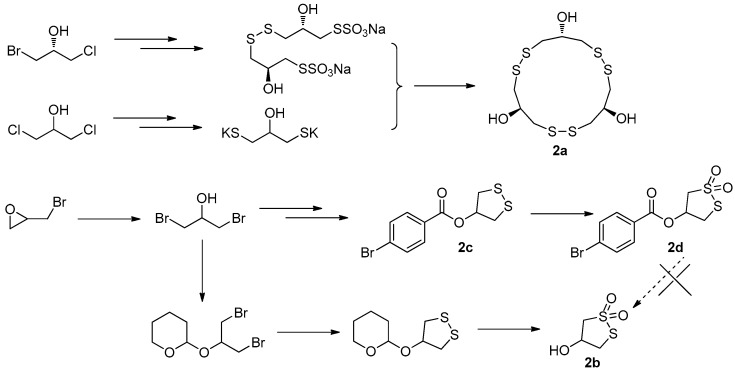
The total synthesis of **2a** and **2b** in Guo’s group [16,18].

The first total synthesis of gymnorrhizol (**2a**) was prepared by Guo’s group in only three steps, starting from (*R*)-1-bromo-3-chloroisopropanol and 1,3-dichloropropan-2-ol as shown in Figure 8. The 1,3-dichloropropan-2-ol was converted into its bispotassium salt, which then reacted with Bunte salt produced from (*R*)-1-bromo-3-chloroisopropanol to give the target compound **2a** in 34% overall yield [16]. Later we started a project to synthesize bruguiesulfurol (**2b**) with epibromohydrin as starting material. As hydroxyl-protecting group, 4-bromobenzoyl was initially selected since it makes small-molecule compound easy to be purified and crystallized. However, the target compound **2b** was not obtained from **2d** in a variety of deprotection conditions, because **2d** cannot be hydrolyzed in acidic conditions and rapidly decomposed in basic conditions. Then, the 2-tetrahydropyranyl group was used as protecting group, which was easily removed under acidic conditions. Finally, the total synthesis of **2b** was successfully achieved for the first time [18], with a key step of mCPBA oxidation and deprotection in one pot with an overall yield of 30% (Figure 8).

**Figure 9:**
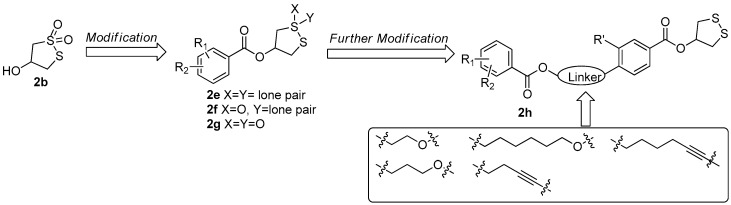
The synthesis of bruguiesulfurol derivatives in Guo’s group [18,20].

During the synthesis of **2b**, a key intermediate **2c** with unoxidized disulfide ring was found to exhibit stronger PTP1B inhibitory activity (IC_50_ = 11.01 µM) than that of **2b** and significant selectivity toward PTP1B versus other PTPs. Based on this observation, a series of 1,2-dithiolane-4-ol derivatives **2e** with different benzoyl group and its oxidative products **2f** and **2g** were prepared [18]. The bioassay results indicated that the compound **2c** still showed the best PTP1B inhibitory and the cyclic dithiolane moiety was essential for PTP1B inhibition. Since the current PTP1B inhibitors usually have undesirable cell permeability and oral bioavailability due to the presence of highly negatively-charged polar pharmacophores in their structures [19], a further structural optimization on **2c** was carried out by the introduction of less polar substituted benzoate in 4-position of benzene with a different aliphatic chain as a linkage (**2h**) aiming at increasing its activity. When the bromine group in **2c** was replaced by the hex-5-yn-1-yl 2,5-dibromobenzoate fragment, the PTP1B inhibitory activity was increased by almost 20-fold (IC_50_ = 0.59 µM) (Figure 9) [20].

### 2.3. Paracaseolide A *(**3**)*

**Figure 10:**
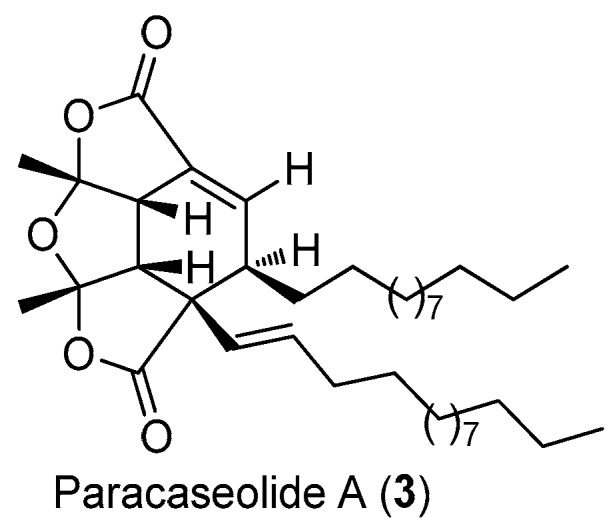
The structure of paracaseolide A (**3**).

Paracaseolide A (**3**), a novel α-alkylbutenolide dimer characterized by an unusual tetraquinane oxa-cage bislactone skeleton bearing two linear alkyl chains, was isolated from the Chinese mangrove plant *Sonneratia paracaseolaris* (Figure 10) [21]. This compound exhibited significant inhibitory activity against dual specificity phosphatase CDC25, a key enzyme for cell cycle progression and associated with tumor aggressiveness, with an IC_50_ value of 6.44 μM.

**Figure 11:**
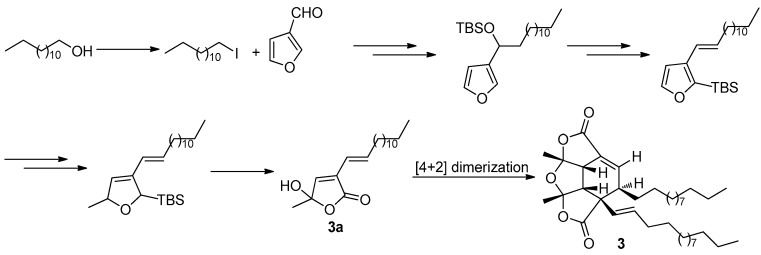
Vassilikogiannakis’s protocol [22] for the synthesis of **3**.

**Figure 12:**
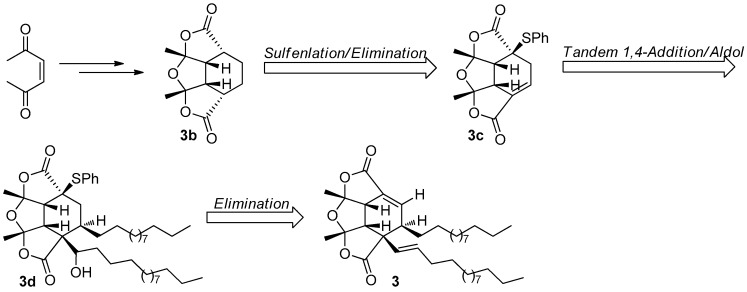
Kraus’s protocol [23] for the synthesis of **3**.

The fascinating, skeletally-unique structure quickly captured the attention of synthetic chemists after a short period of its reveal. The first total synthesis of paracaseolide A (**3**) was reported by Noutsias and Vassilikogiannakis [22]. The synthetic strategy was based on a bioinspired [4 + 2]-dimerization/ketalization/epimerization of a 4-hydroxybutenolide precursor **3a**, generated by singlet oxygen-mediated oxidation of a furan intermediate formed from tridecan-1-ol and furan-2-carbaldehyde (Figure 11). In order to obtain suitable quantities of **3** for SAR study, Kraus and Guney developed a concise synthetic route allowing strategically-distinct generation of **3** and its analogs [23]. Their syntheses began with ene-2,5-dione, which was converted into the key tetraquinane oxa-cage bis-lactone **3b** characterized by a great potential to be introduced functionality into its molecular skeleton. Finally, the target **3** was synthesized after a tandem vicinal difunctinalization of a α,β-unsaturated lactone **3c** to form **3d**, and followed by elimination to achieve the requisite unsaturation (Figure 12). In addition, researchers from other five different groups have also reported the total synthesis of **3** involving the same key step as that of Vassilikogiannakis’s protocol, namely the [4 + 2]-dimerization of 4-hydroxybutenolide (**3a**) [24,25,26,27,28].

### 2.4. (R)-de-O-Methyllasiodiplodin *(**4**)*

**Figure 13:**
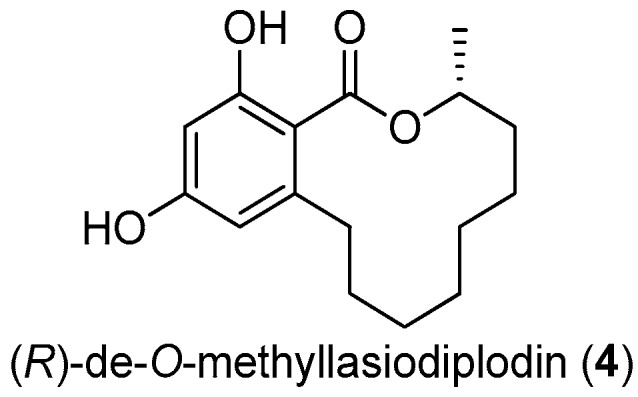
The structure of (*R*)-de-*O*-methyllasiodiplodin (**4**).

The twelve-membered macrolide (*R*)-de-*O*-methyllasiodiplodin (**4**, Figure 13), previously isolated from plants and fungus, was found to show various bioactivities, such as inhibition of prostaglandin biosynthesis, antimicrobial, and significant antitumor activities [29]. This macrolide was found to be a potent nonsteroidal antagonist of mineralocorticoid receptor (MR), a validated target for treating hypertension and other cardiovascular diseases, with an IC_50_ value of 8.93 μM [30].

**Figure 14:**
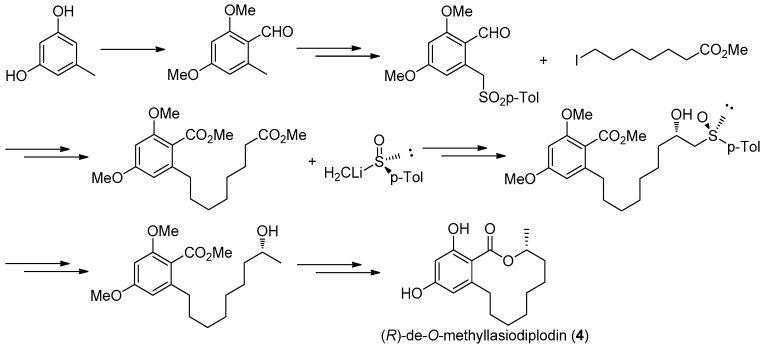
Ruano’s protocol [31] for the synthesis of **4**.

**Figure 15:**
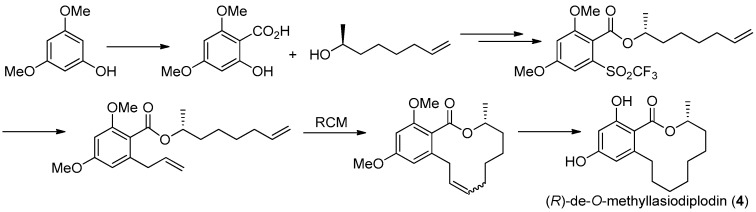
Alois Fürstner’s protocol [32] for the synthesis of **4**.

**Figure 16:**
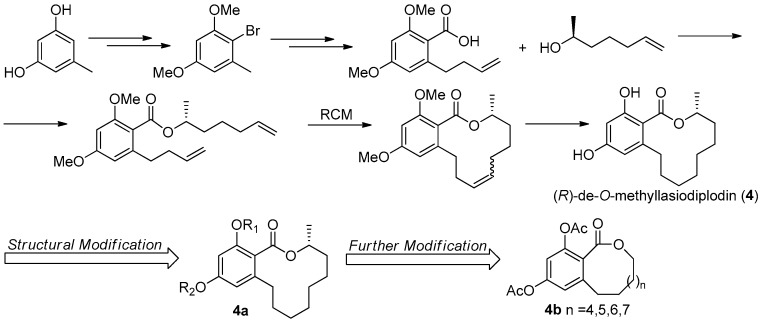
Synthesis and structural modification of **4** in Guo’s group [30,33].

The first asymmetric total synthesis of **4** was published in 1990 by Ruano et al. [31]. The orcinol monohydrate was used as a starting material and the key chiral center was smoothly created in the last synthetic steps by asymmetric induction of a chiral sulfoxide group as shown in Figure 14. However, this synthetic route was somewhat complicated and low-yielding with 18 steps in 0.8% overall yield. In 1996, a short and flexible route toward **4**, starting with 3,5-dimethoxyphenol which was subsequently carboxylated via a kolbe-Schmitt reactions, was developed by Alois Fürstner and coworkers [32]. Finally, the macrolide ring was formed in the key ring closing metathesis (RCM) reaction catalyzed by Gurbbs I generation catalyst (Figure 15). In 2009, a more facile and efficient preparation for **4** suitable for the general synthetic laboratories was completed [33]. The synthetic route to **4** shown in Figure 16 was similar with Fürstner’s protocol, but started from orcinol monohydrate and used Gurbbs II catalyst in the RCM reaction. Noticeably, the yield of the last demethylation step was increased to 57% by performing the quenching under reduced pressure, compared with Ruano’s procedure (17% yield).

To optimize the activity of **4**, a series of hydroxyl-derivatives **4a** were prepared and evaluated for their antagonistic activity against MR. The bioassay result indicated that the diacetylated compound exhibited a more potent antagonistic effect against MR that **4**, with an IC_50_ value of 2.78 μM. Further, another series of derivatives were prepared by preserving diacetlylate moiety and introducing different ring size of the lactone without chiral methyl. Finally, the IC_50_ value for MR antagonistic activity was increased to 0.58 μM, when the n was 5. The current SAR study indicated that the acetylation at the phenolic hydroxyl group increases the MR antagonistic effect and the ring size of the lactone was very crucial for its activity [30].

### 2.5. Methyl Spongoate *(**5**)*

**Figure 17:**
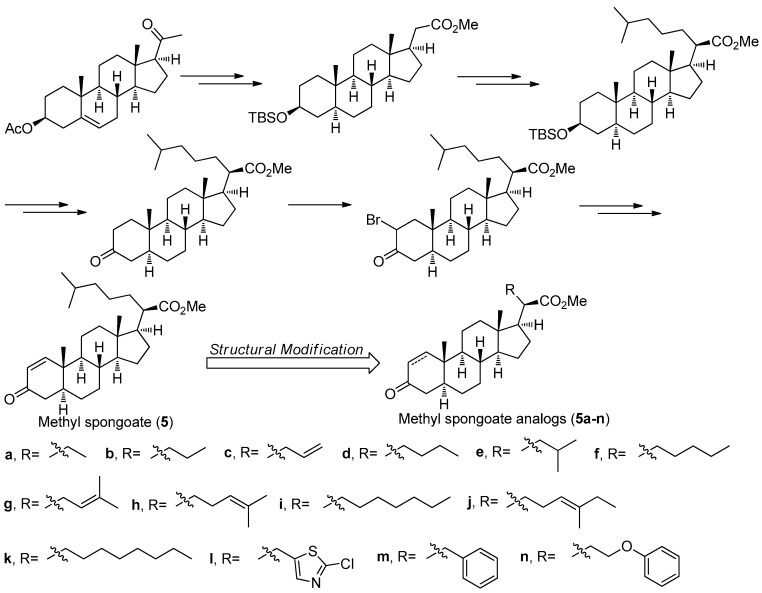
The structure, synthesis and SAR study of **5** in Guo’s group [35,36].

Methyl spongoate (**5**) (Figure 17) is a novel steroid with an uncommon C-20 methoxycarbonyl group with *R* absolute configuration, which was isolated from the Sanya soft coral *Spongodes* sp. This compound showed significant cytotoxicity against BEL-7402 (IC_50_ = 0.33 μM), P388 (IC_50_ = 8.9 μM), A-549 (IC_50_ = 11.7 μM), and HT-29 cell lines (IC_50_ = 11.7 μM) [34]. However, its low natural yield with 2.5 mg obtained from 456 g (dry weight) of the *Spongodes* sp. limited further potential pharmacological studies. In order to supply sufficient amount of **5**, its first stereoselective synthesis was carried out by Gong et al. [35]. As shown in Figure 17, the total synthesis of **5** started from the pregnenolone acetate and was successfully achieved in 14 steps with an 11% overall yield, which is amenable to scale-up in an average chemical laboratory.

Based on a similar synthetic strategy, a series of related derivatives **5a-n** with different C-20 side-chains were prepared for further anti-tumor SAR studies [36]. All of the analogs and **5** were evaluated against a panel of cancer cell lines including A549, HCT-116, HepG2, SW-1990, MCF-7, and NCI-H460. The results showed that compound **5i** having a heptyl side chain had the best inhibition against HepG2, SW-1990, MCF-7, and NCI-H460 cell lines with IC_50_ values ranging from 6 to 10 μM, much better that that of **5** (IC_50_ values ranging from 17 to 30 μM). The preliminary SAR study not only have demonstrated that the side-chain has an important role in determining antitumor activity but also confirmed the significance of the α,β-unsaturated carbonyl moiety of A ring in the steroid as an electrophilic Michael acceptor in the antitumor activity.

### 2.6. Methyl Xestospongoate *(**6**)* and Distaminolyne A *(**7**)*

**Figure 18:**
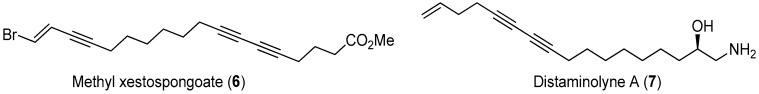
The structures of methyl xestospongoate (**6**) and distaminolyne A (**7**).

Polyacetylenic natural products isolated from marine algae and invertebrates often displayed a broad array of biological properties, including cytotoxicity, antimicrobial activity, and various enzyme inhibitory activities [37]. During the investigation of components of the extract of marine sponge *Xestospongia testudinaria*, methyl xestospongoate (**6**, Figure 18), a brominated polyacetylenic fatty acid, was found to exhibit strong pancreatic lipase (PL) inhibitory activity with an IC_50_ value of 3.1 µM [38,39].

**Figure 19:**
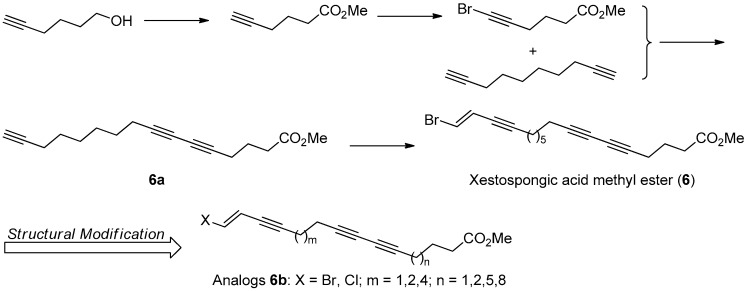
The synthesis and structural modification of **6** in Guo’s group [40].

The first total synthesis of **6** was achieved in five steps with 30% overall yield, based on the retrosynthesis analysis. The key synthetic steps include the Sonogashira coupling reaction of 1,2-dibromoethylene with intermediated **6a**, and the Cu(I) catalyzed cross-coupling reaction of 1,9-decadiyne with methyl 6-bromo-5-hexynate (Figure 19).

To better understand its SAR information, a following structural modification on **6** was carried out to produce a series of halogenated analogs **6b** with different chain length. The bioassay results indicated that the PL inhibitory activity of the terminal brominated ones are better than that of the chlorinated ones, and the chain length with 16-20 C-atom might be the most optimal for their PL inhibitory activity. However, the structural modification made activity of all these analogs decrease or lose, with inhibition percentage at 50 µM less than 44.53%, compared with that of **6** with 68.84% inhibition at 50 µM [40].

**Figure 20:**
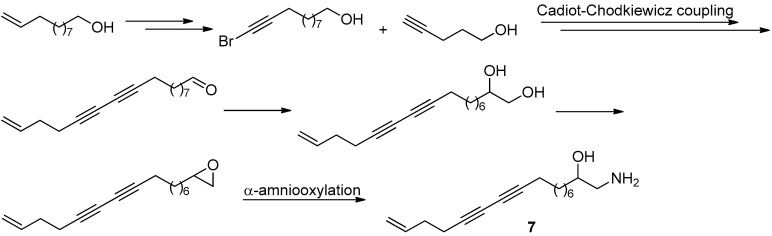
The synthesis of **7** in Guo’s group [42].

Distaminolyne A (**7**, Figure 18) was an antibacterial acetylenic amino alcohol isolated from New Zealand ascidian *Pseudodistoma opacum* [41]. The structure of this compound is similar to that of methyl xestospongoate (**6**), making it a possible PL inhibitor. In addition, the chiral 1,2-amino alcohol group is an important structural function group in bioactive compounds. All these observations led us to embark on the total synthesis of distaminolyne A (**7**) and its enantiomer (**7a**). The first total synthesis of both compounds were achieved from the commercially-available undec-10-en-1-ol (Figure 20) [42]. A key proline-catalyzed asymmetric α-amino-oxylation of an aldehyde intermediate was used to introduce the chiral center en route to the enantiomerically-pure 1,2-amino alcohols. Bioassay result revealed that both enantiomers displayed weak PL inhibitory activity, with 36.7% and 24.7% at 50 µM, respectively. Interestingly, the [α]_D_ values of both synthesized enantiomers indicated that the absolute configuration of the natural distaminolyne A should be revised as 2*R*.

## 3. Conclusions

Marine natural products (MNPs) have become a valuable resource for the development of novel drugs. However, their low natural yield extremely limits the further druggability research and few of them can be directly used in clinical applications. Therefore, the total syntheses of MNPs are essentially necessary for studying their biological activities, while the structural modifications are useful for yielding safer, more potent and selective molecules, with improved physicochemical and pharmacokinetic properties. The examples above briefly illustrate the synthesis and structural modification of some selected MNPs, which is only a small basic part during the development of marine product-derived drugs in recent years. To promote the development of efficient synthetic and modified strategies for MNPs, closer cooperation between organic chemists, medicinal chemists, and pharmacologists is desired.
